# A circuitry for sleep in Parkinson's disease

**DOI:** 10.18632/oncotarget.14000

**Published:** 2016-12-16

**Authors:** Adriano D.S. Targa, Marcelo M.S. Lima

**Affiliations:** ^1^ Laboratory of Neurophysiology, Department of Physiology, Biological Sciences Division, Federal University of Paraná, Curitiba, Brazil

**Keywords:** Parkinson's disease, REM sleep, dopamine, premotor, Neuroscience

Parkinson´s disease (PD) is a neurodegenerative condition that usually begins in the fifth or sixth decade of life with an overall crude prevalence (including males and females across the entire age range) between 100 and 200 per 100 000 persons [1]. Considering the United Nations projections, the number of people aged 65 or older in 2010, estimated in 524 million, will grow to nearly 1.5 billion in 2050, with most of the increase in developing countries [2]. With this scenario, it is expected an increasing number of PD cases worldwide that invariably will be associated with movement disorders including asymmetric onset of bradykinesia, rigidity, rest tremor and disturbances in balance. Such afflictions were originally described 200 years ago by James Parkinson, and are, still today, the classical features for the clinical diagnosis.

However, a multitude of premotor, that is, early-phase disturbances, have been recently and richly described as neuropathological hallmarks of the disease progression serving as new diagnostic assessments. In view of that, sleep disturbances usually emerge early in the disease course as a product of the degeneration of non-dopaminergic regions, like pedunculopontine tegmental nucleus (PPT), lower raphe nuclei and locus coeruleus [3]. Besides, the occurrence of a dopaminergic lesion within the substantia nigra pars compacta (SNpc), typically impacting in motor disability, is also reported to promote a selective impairment in the generation and maintenance of rapid eye movement (REM) sleep [4]. Also, the PPT and the basal ganglia share substantial amount of anatomical and functional similarities. Among these, there are reports of a role in locomotion, memory consolidation and sleep regulation [5, 6]. Therefore, the conjuncture of elements described at that time engaged us to propose a hypothesis aiming to explain sleep disturbances in PD based on the degeneration of the reciprocal connections between PPT and SNpc during the evolution of the disease [7].

Accordingly, our recent study published in *Neuropharmacology* proposed a physiological association between PPT and SNpc that was tested by a protocol of neurotoxic lesions and pharmacological modulation of striatal D2 receptors in Wistar rats [8]. These results showed that D2 striatal receptors have a relevant influence in non-REM sleep regulation because raclopride (a selective antagonist) administration increased the percentage of time spent in non-REM sleep compared to piribedil (a selective agonist). We demonstrated that a punctual PPT lesion (inflicted by ibotenic acid) blocked the compensatory manifestation of REM sleep, after 24 h of REM sleep deprivation. However, this effect was prevented when the animals were treated with intra-striatal piribedil. Thus, these results showed that striatal D2 receptors activation reinforced the pathway and, consequently, prevented sleep disturbances, when cholinergic tone within PPT was decreased.

The SNpc lesion (inflicted by the neurotoxin rotenone) prevented the manifestations of REM sleep rebound and this effect was more intense than the ibotenic acid induced lesions, since the striatal activation did not reverse it. Furthermore, the most striking result was that rotenone lesion decreased the time spent in non-REM sleep. Moreover, we found a positive correlation between the percentage of time spent in non-REM sleep and dopamine levels within the SNpc and striatum in the sham REM sleep deprived animals. Hence, we suggested that dopamine is a keyplayer in non-REM sleep regulation. Such result was unexpected, since dopamine was mostly associated with REM sleep. Thus, the occurrence of non-REM sleep deficits, perhaps prior to REM sleep dysfunction, could be an important clue for novel diagnostic strategies in PD.

Our findings, collectively, suggest a new circuitry for sleep regulation in PD, involving the so called triad of nuclei composed by PPT, SNpc and striatum, evidencing not only a potential therapeutic target for the sleep disturbances associated to this pathology, but also a remarkable opportunity of premotor diagnosis. Since originally described by Parkinson in 1817, a lot was accomplished regarding diagnosis and treatment, but certainly the last 15 years were accompanied by the most extraordinary advances, galvanizing sleep disturbances as the main source of prodromal markers.
